# **Excitation functions of** ^72^**Ge(***p***,x***n***)**^72,71^**As reactions from threshold up to 45 MeV for production of the non-standard positron emitter **^**72**^**As**

**DOI:** 10.1038/s41598-024-67319-9

**Published:** 2024-07-19

**Authors:** Mazhar Hussain, Lucas Mues Genannt Koers, Ingo Spahn, Stefan Spellerberg, Bernd Neumaier, Syed M. Qaim

**Affiliations:** 1 Institut für Neurowissenschaften und Medizin, INM-5: Nuklearchemie, Forschungszentrum Jülich (FZJ), 52425 Jülich, Germany; 2 Department of Physics, Government College University Lahore (GCUL), Lahore, 54000 Pakistan; 3 Institute of Radiochemistry and Experimental Molecular Imaging, University of Cologne, Cologne, 50937 Germany

**Keywords:** Chemistry, Physics

## Abstract

Nuclear reaction cross sections for the formation of ^72^As and ^71^As in proton-induced reactions on enriched ^72^Ge targets were measured up to 45 MeV utilizing three different cyclotrons at the Forschungszentrum Jülich. The stacked-thin sample activation technique in combination with high-resolution γ-ray spectrometry was used. The major *γ*-ray peaks of ^72^As and ^71^As formed via the ^72^Ge(*p*,n)^72^As and ^72^Ge(*p*,2*n*)^71^As reactions, respectively, were analyzed. The incident proton energy and flux on a foil were determined using several monitor reactions. Based on integrated counts, irradiation data and the nuclear decay data, the reaction cross sections were measured. All data describe the first measurements. Theoretical nuclear model calculations were then carried out by using the codes TALYS 1.96, EMPIRE 3.2 and ALICE-IPPE. A very good agreement between the measured data and calculated values was found. The new data enabled us to calculate the thick target yields and estimate the radionuclidic impurities for a given energy range. Over the optimum energy range Ep = 14 → 7 MeV, the calculated thick target yield of ^72^As amounts to 272 MBq/μAh with no ^71^As impurity at all. The ^72^Ge(*p*,n)^72^As reaction on the enriched ^72^Ge is thus very suitable for clinical scale production of ^72^As at a medical cyclotron.

## Introduction

Arsenic is an element of relevance to environmental and toxicological sciences. In addition several radioisotopes of arsenic are very promising for applications in the fields of pharmacology, diagnostics and cancer treatment^1–4^. Of considerable present interest is the theranostic approach, which entails the use of two radionuclides of the same element in identical chemical form, one a positron emitter for measuring the distribution kinetics of the radioactivity in the body via Positron Emission Tomography (PET), and the other a radionuclide emitting corpuscular radiation (β^˗^, α or Auger electrons) useful for internal radiotherapy^5^. The two radionuclides are denoted as “matched pair”^5,6^. A detailed review of production methodologies of the commonly used matched-pairs, e.g. ^44^Sc/^47^Sc, ^64^Cu/^67^Cu, ^86^Y/^90^Y,^124^I/^131^I, etc. has been published^6^. Such a pair exists also in the case of arsenic, namely ^72^As/^77^As, which has so far not been much investigated but which is of considerable potential interest in radiotheranostics using antibobodies^6–8^. The radionuclide ^77^As is a β^˗^-emitter (T_1*/*2_ = 38.8 h; E_maxβ˗_ = 0.7 MeV, I_β˗_ = 100%) and is of therapeutic interest. It is produced in a nuclear reactor in a no-carrier-added form via the route ^76^Ge(n,γ)^77 m,g^Ge $\to ^{{{\upbeta } - }}$ ^77^As, and the production methodology has been well developed^9,10^. The diagnostic partner ^72^As, on the other hand, is a β^+^-emitter (T_1/2_ = 26.0 h; E_maxβ+_  = 1.17 MeV, I_β+_  = 87.8%) which can be produced only at a cyclotron. The relevant production methodologies for this radionuclide need considerable development.

There appear to be three potentially useful production routes for ^72^As:

- ^72^Se/^72^As generator system
- ^72^Ge(p,n)^72^As
- ^69^ Ga(α,n)^72^As

The development work involves several facets. We, however, limit ourselves to reaction cross-section data. A critical analysis of the nuclear data available in the literature (EXFOR)^11^ revealed that some data for the ^75^As(p,4n)^72^Se reaction, commonly used for the production of the generator parent ^72^Se are available. Experimental cross sections up to 45 MeV were found consistent^12,13^. However, the recent measurements in the high energy region significantly deviate from each other^14,15^. A critical analysis of this reaction has also confirmed that data are discrepant^16^.

The data for the other two reactions are deficient. At the Forschungszentrum Jülich we have started an extensive programme of detailed cross section measurements of the above-mentioned routes using three cyclotrons. In this work we report on our studies on the ^72^Ge(p,n)^72^As reaction using a highly enriched target.

Cross section measurements for the production of ^72^As using enriched ^72^Ge have to date not been properly performed. In literature, only an old report by Levkovskii^17^ is found, but without any experimental details. We discuss that work in the relevant section. Some laboratories have tried to produce ^72^As by using natural germanium^18–21^. But the use of natural germanium as a target results in a large number of nuclear reactions that lead to the formation of a bunch of impurities in the product. Therefore, all those previously carried out works are only of limited use with regard to clean production of ^72^As. On the other hand, even while using enriched ^72^Ge as target, in the study of proton induced reactions at higher energies, ^71^As (T_1/2_ = 65.30 h), ^70^As (T_1/2_ = 52.6 min) and several isotopes of Ge and Ga are also formed as radionuclidic impurities. Whereas the Ge and Ga isotopes can be chemically removed, the impurity levels of As isotopes can be controlled only through a proper knowledge of nuclear data. Therefore, we measured cross sections for the production of both ^72^As and ^71^As from ^72^Ge up to 45 MeV. The isotopic composition of the enriched material and the associated chemical impurities are given in Table 1 as certified by the supplier. The decay data of ^77^As, the expected reaction products and the Q-values are given in Tables 2 and 3, respectively^22^.

**Table 1:** Isotopic composition of the enriched target material and the level of chemical impurities.

Isotope	Isotopic composition [%]	Chemical impurity	Level [ppm]
Ge-70	0.49	Co	< 0.001
Ge-72	96.42	Cd	< 0.003
Ge-73	2.94	Fe	0.015
Ge-74	0.145	Mn	< 0.003
Ge-76	0.005	Cr	< 0.003
		Al	0.01
		Cu	< 0.003
		Zn	< 0.003
		Ca	< 0.01
		Si	0.01
		Mg	< 0.003
		Mo	< 0.001
		Ni	< 0.005

**Table 2:** Decay data of ^77^As and the radionuclides identified in the irradiated ^72^Ge samples together with the radionuclides formed in the monitor foils of ^nat^Cu and ^nat^Ti.

Radionuclide	Mode of decay[%]	Half-life	E_*γ*_ (keV)	I_*γ*_ [%]
Investigated reaction products				
^72^As	β + (87.8)	26.0 h (1)	629.92 (5)	8.07 (24)
	EC (12.2)		834.99 (3)	81.0 (20)
^71^As	β + (28.4)	65.3 h (1)	174.95 (5)	82.45 (22)
	EC (71.6)		326.785 (15)	3.05 (9)
			499.876 (10)	3.64 (10)
			1095.49 (10)	4.11 (12)
			139.46 (19)	0.804 (22)
^77^As	β^˗^ (100)	38.8 (5)	239.01 (6)	1.59
			249.81 (8)	0.39 (6)
Monitor reaction product				
^62^Zn	β + (8.2)	9.19 h (15)	548.35 (11)	15.3 (14)
	EC (91.8)		596.56 (13)	26.0 (20)
^63^Zn	β + (92.7)	38.47 min (5)	669.62 (5)	8.2 (3)
	EC (7.3)		962.06 (4)	6.5 (4)
^65^Zn	β + (1.4)	243.93 d (9)	1115.4 (2)	50.04 (10)
	EC (98.6)			
^58^Co	β + (14.9)	70.86 d (6)	810.76 (20)	99.45 (1)
	EC (85.1)			
^48^ V	β + (50.4)	15.97 d(3)	983.53 (4)	99.98 (4)
	EC (49.6)		1312.11 (6)	98.2 (3)
^46^Sc	β^˗^ (100)	83.79 d (4)	889.28 (3)	99.98 (1)
			1120.55 (4)	99.99 (1)

The data are taken from the NuDat 3.0 database of the National Nuclear Data Center, BNL, USA^22^.

**Table 3:** Threshold energies of the reactions to produce ^71^As, ^72^As, ^62^Zn, ^63^Zn, and ^65^Zn, as calculated from Q-value calculator^22^.

Product nuclide	Nuclear reaction	Q-value (MeV)	Threshold energy (MeV)
^72^As	^72^Ge(*p*,*n)*	− 5.1384	5.2104
^71^As	^72^Ge(*p*,2*n)*	− 13.5463	13.7361
^70^As	^72^Ge(*p*,3*n)*	− 25.1769	25.5296
^65^Zn	^65^Cu(*p*,*n)*	− 2.1340	2.1671
^63^Zn	^63^Cu(*p*,*n)*	− 4.1488	4.2152
^62^Zn	^63^Cu(*p*,2*n)*	− 13.2654	13.4778

Besides obtaining data for optimization of ^72^As-production via the ^72^Ge(p,n)-reaction, a strong motivation in this work was to apply vigorous tests to nuclear model calculations, a prerequisite of which is the availability of accurate experimental data on monoisotopic or enriched targets to limit the number of contributing processes to the formation of a desired product^23^. We compared our new data with global model calculations in the case of the codes ALICE-IPPE^24^ and EMPIRE 3.2^25^. In the case of TALYS 1.96^26^, however, we compared our data with TENDL-2021^27^ which is based on global parameters but also performed a detailed parametrization and analysis. The quality of the agreement between experiment and theory considerably improved with the choice of proper input parameters.

### Comparison of experimental and theoretical excitation function

#### ^72^Ge(*p*,*n*)^72^As reaction

**Figure 1:**
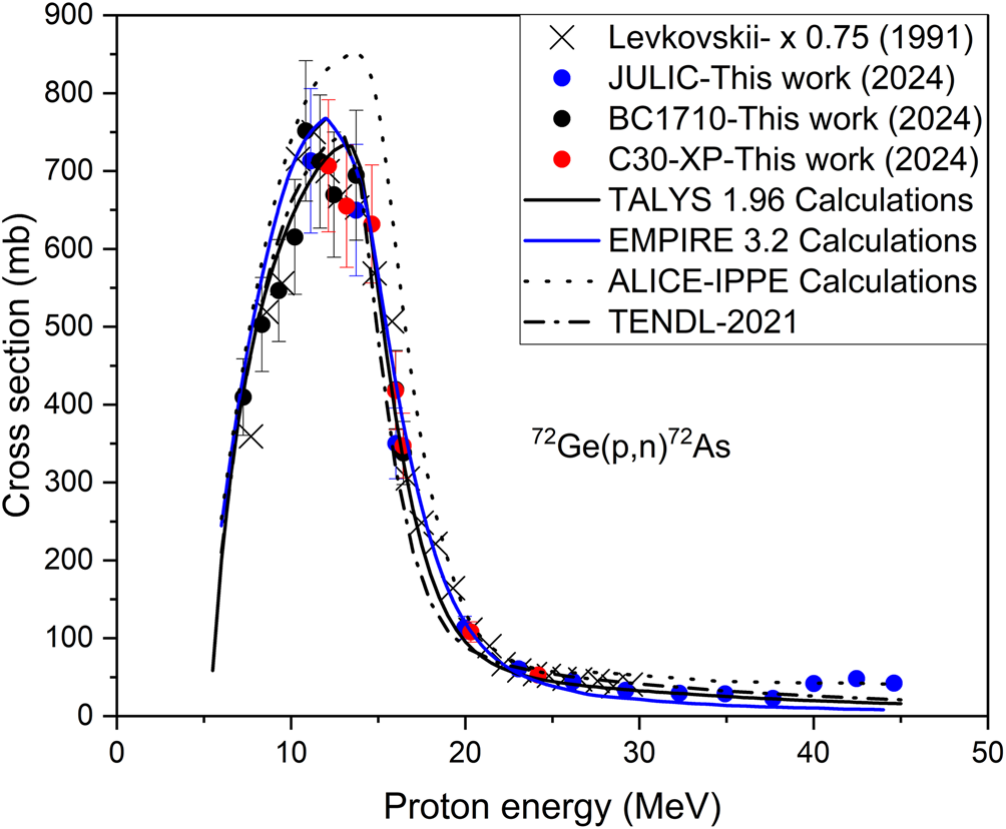
Measured and calculated excitation function of the ^72^Ge(*p*,*n*)^72^As reaction up to 45 MeV proton energy.

The excitation function of the ^72^Ge(*p*,n)^72^As reaction measured in this work is shown in Fig. 1. The data obtained using the three cyclotrons (JULIC, BC1710, C30XP)) are given in different coulours. The calculated cross sections by using the codes TALYS 1.96^26^, EMPIRE 3.2^25^ and ALICE-IPPE^24^ are also given in the same figure. It is observed that the measured values are generally in good agreement with the calculational results. The results achieved by ALLIC-IPPE are slightly higher but within the limits of the uncertainties. Levkovskii^17^ reported some raw data for this nuclear reaction without the details of enrichment of the target material and the decay data. It has been shown that those data are wrong and should be lowered by 25% to account for the wrong monitor reaction cross section used^28^. Therefore, we plotted the corrected data in Fig. 1. The normalization factor was deduced from the cross sections of the monitor reactions. Those normalised data of Levkovskii are in good agreement with our measurements except for a lower trend in the optical model part of the excitation function. Considering the total uncertainty, both the experimental and theoretical values are within the limits of error bars. Surprisingly, even the global calculation TENDL-2021 describes the excitation function fairly well. The pre-equilibrium part of the excitation function was thus successfully measured and TALYS 1.96 results reproduced the data very well.

#### ^72^Ge(*p*,2*n*)^71^As reaction

**Figure 2:**
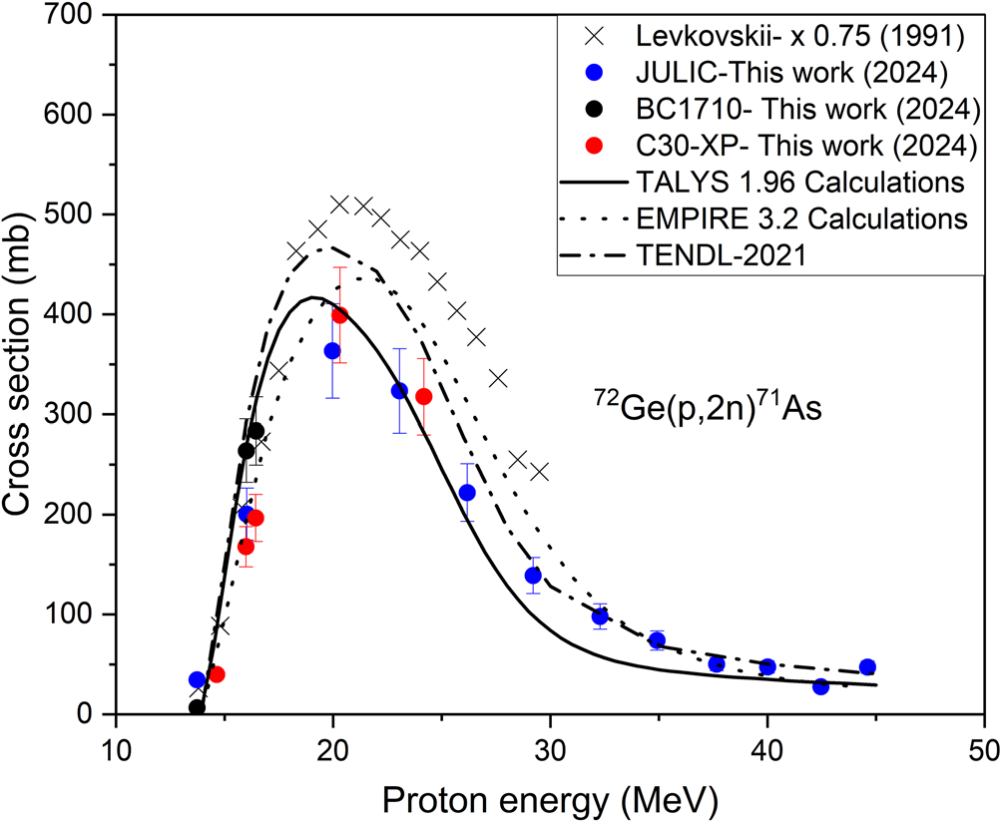
Measured and calculated excitation function of the ^72^Ge(*p*,*2n*)^71^As reaction up to 45 MeV proton energy.

The measured excitation function for the ^72^Ge(*p*,2n)^71^As reaction is shown in Fig. 2 together with the calculated cross sections by using the codes TALYS 1.96 with adjusted parameters as well as the code EMPIRE 3.2. It is observed that the measured values are in good agreement with the calculational results of TALYS 1.96 except for the values in the energy region above 28 MeV. In that region, the TENDL-2021 values, which are based on calculations using global parameters, are in better agreement with our experimental data. The EMPIRE 3.2 nuclear model calculation shows a higher trend in the excitation function. Levkovskii reported some raw data for this nuclear reaction as well. Those data were about 40% higher than our measurements. We normalised those data by 25% (as mentioned above) but they still fall out of our measurements and the calculated results. All their cross section values between 18 MeV and 30 MeV are higher than our data and the results of TALYS 1.96 could also not reproduce those data. We are therefore of the view that those data should be discarded.

### Thick target yields and production possibility

**Figure 3:**
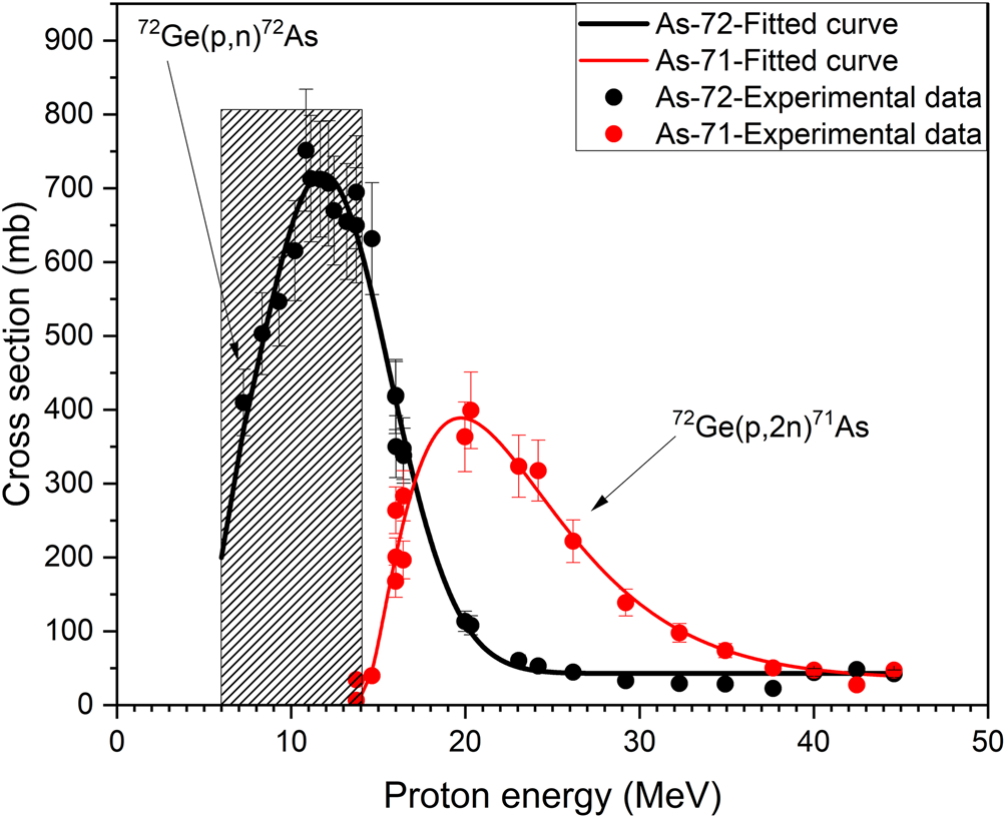
Our measured data points and fitted curves to describe the excitation functions of the ^72^Ge(*p*,*xn*)^71,72^As reactions up to 45 MeV proton energy.

**Figure 4:**
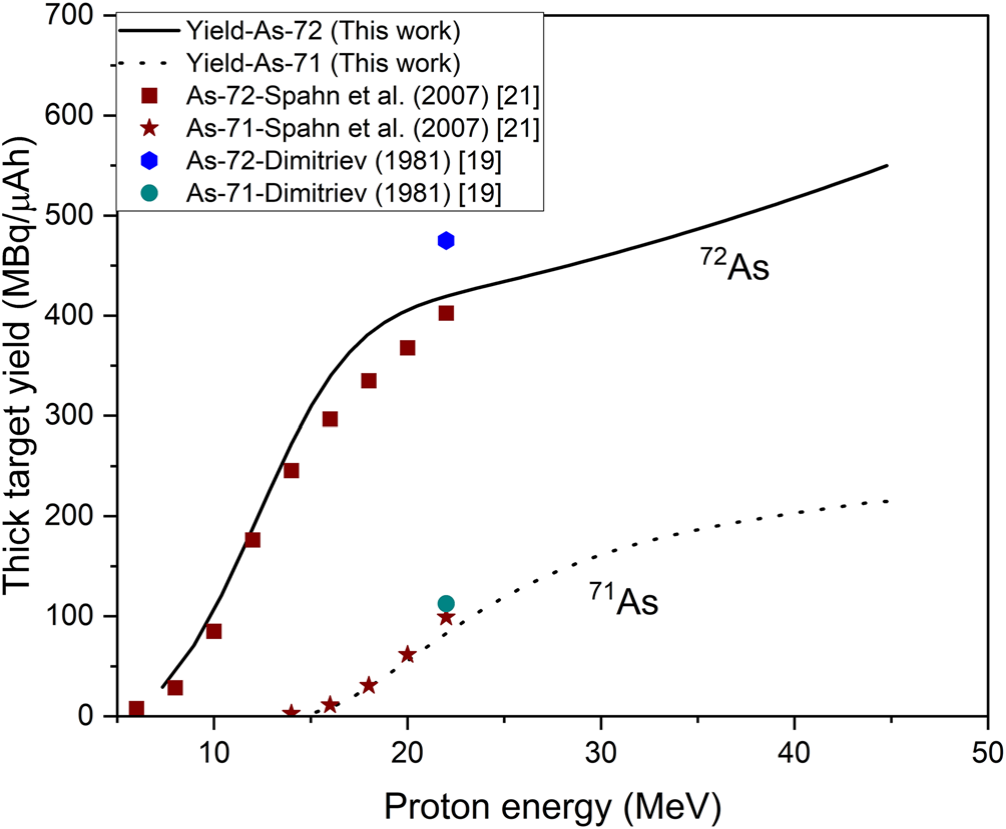
Thick target yields calculated from the fitted excitation functions of ^72^Ge(p,n)^72^As and ^72^Ge(p,2n)^71^As reactions leading to the production of ^72^As and ^71^As, respectively. The literature data ^19,21^ normalised to the enrichment of ^72^Ge are also shown.

For practical application we relied more on our experimental data. All the measured data were fitted by using the basic fitting functions integrated in the software ORIGIN Pro^29^. The result is shown in Fig. 3. From the excitation functions, thick target yields of the two radionuclides, namely ^71^As and ^72^As were calculated using the well-established equations^30,31^ and assuming an irradiation time of 1 h and the proton beam current as 1 μA. The results are shown in Fig. 4 as a function of proton energy. The results of two previous works^19,21^ are also depicted in the same figure. Evidently, the integral yields of ^72^As are higher than ^71^As in all energy regions. The results of a comparative analysis of some selected energy ranges are collected in Table. 4. It should be interesting to compare the yields calculated from the excitation functions with those measured experimentally using a thick target. Dmitriev and Molin^19^ reported yields for 22 MeV protons on ^nat^Ge. The natural abundance of ^72^Ge is 27.45%; whereas the contribution in the enriched material it is more than three times higher. We extrapolated their yield data to 96.4% enrichment (enriched target of this work) and presented the result in Fig. 4 for comparison. Similarly the yields reported by Spahn et al.^21^ for ^nat^Ge were also extrapolated (see Fig. 4). Spahn et al.^21^ estimated a thick target yield of 114.1 MBq/μAh for 22 MeV protons on ^nat^Ge. A comparison of those yields with our yields for the same proton energy showed that our values are higher than those data. An extrapolation of their work to the 96.4% enrichment of ^72^Ge led to a good agreement between the two data sets.

**Table 4:** Thick target yields and impurity level in the production of ^72^As using 96.4% enriched ^72^Ge target.

Energy range (MeV)	Thick target yield (MBq/µAh)^72^As	Thick target yield (MBq/µAh)^71^As	Percentage impurity % (^71^As)	Percentage impurity % (^73^As)	Percentage impurity % (^74^As)
14 → 7	272.1	0.0	0.0	˂ 0.05	˂ 0.01
15 → 7	310.3	2.7	0.8	˂ 0.05	˂ 0.01
16 → 7	340.7	8.8	2.6	˂ 0.05	˂ 0.01
18 → 7	380.8	28.1	7.4	˂ 0.05	˂ 0.01
22 → 7	419.7	83.8	19.9	˂ 0.05	˂ 0.01
25 → 7	434.1	119.2	27.5	˂ 0.05	˂ 0.01
22 → 7 (Spahn et al. 2007) using ^nat^Ge	114.1	28.1	24.6		
22 → 7 (Dimitriev 1981) using ^nat^Ge	135	32	23.7		

From the viewpoint of clean production of ^72^As, it is necessary to estimate the amount of longer-lived ^73^As and ^74^As also in the chosen energy range. Therefore, we have calculated the thick target yields of both those radionuclides from the model-calculated results and converted the data according to the enrichment composition of our samples. The results are given in Table 4. Regarding the production possibilities of ^72^As, a few suitable energy ranges and estimation of the radionuclidic impurity ^71^As are also given in Table 4. To achieve a high purity ^72^As for medical applications, a low energy range 14 → 7 MeV is recommended where no ^71^As is formed. From the yield curves given in Fig. 6, it is obvious that the radionuclide ^72^As can also be produced in large amounts at cyclotrons with energies up to 45 MeV. However, it will contain a considerable proportion of the somewhat longer-lived ^71^As. Regarding some impurity radionuclides of Ge and Ga (e.g. ^71^Ge, ^69^Ge, ^68^Ga, ^67^Ga, etc.), possibly formed in proton irradiations of enriched ^72^Ge, a solvent extraction separation method developed in our Institute^32^ would lead to high-purity radioarsenic.

## Conclusions

Experiments were carried out at three different cyclotrons to measure the formation of ^72^As and ^71^As up to 45 MeV proton energy. Individual nuclear reaction cross sections for the processes ^72^Ge(p,n)^72^As and ^72^Ge(p,2n)^71^As were obtained for the first time. The modern nuclear reaction modeling codes, namely TALYS 1.96, EMPIRE 3.2 and ALICE-IPPE were employed to get the theoretical results of excitation functions. An agreement between the measured data and theoretical results was obtained after choosing proper input parameters. From the fitted curves, the thick target yields were calculated. Therefrom, optimum conditions for the production of the non-standard positron emitter ^72^As were deduced. A proton energy range of 14 → 7 MeV on an enriched ^72^Ge target is recommended for its production with minimum impurity level of ^71^As. The method appears to be very suitable for clinical scale production of ^72^As at a medical cyclotron because a simple solvent extraction separation procedure for radioarsenic has also been developed.

## Methods and analytical procedures

### Samples and irradiations

**Figure 5:**
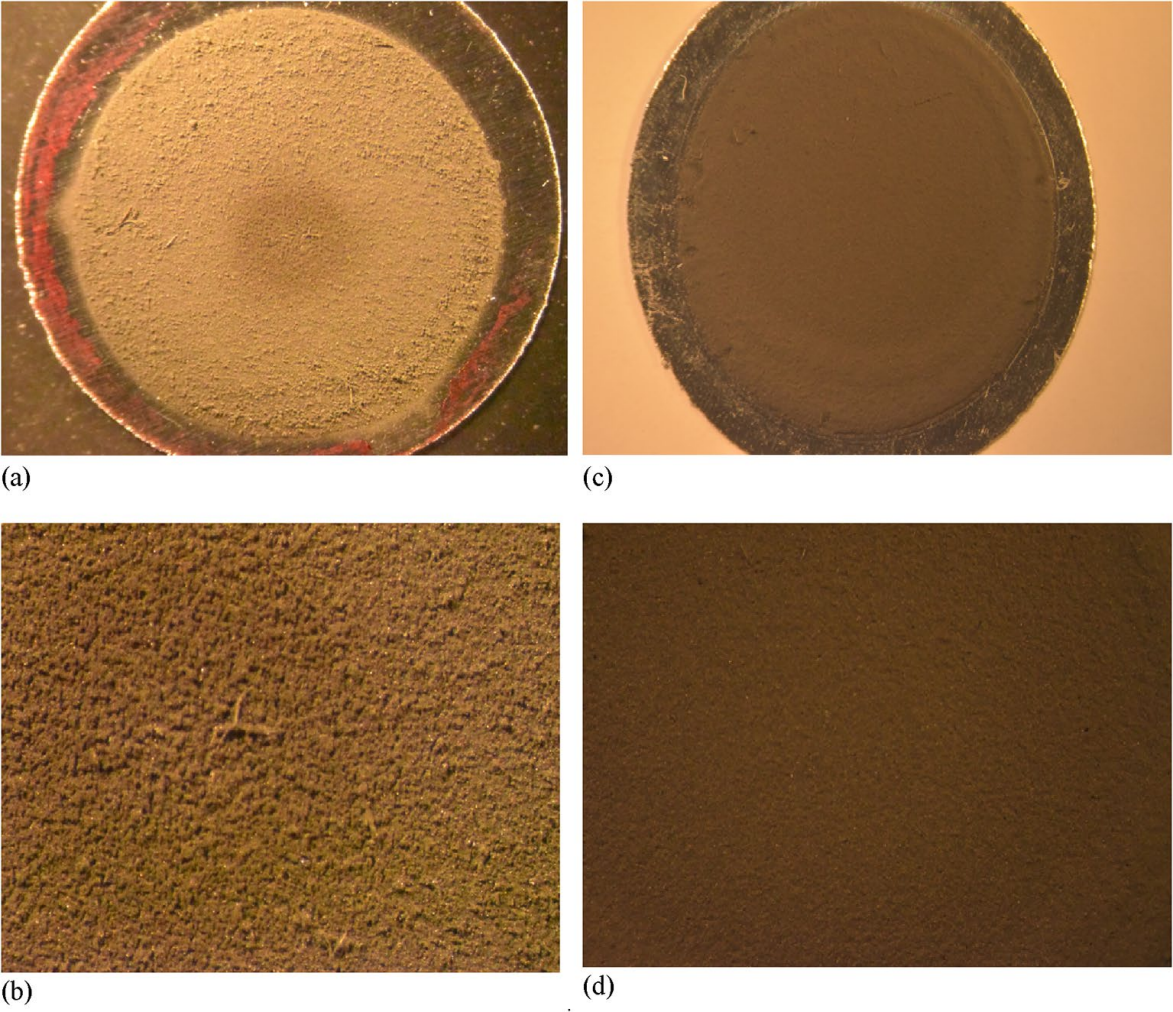
The enriched germanium target developed on aluminum backing without cover. (**a**) Sample 14 showing a thin sediment on Al backing. (**b**) Magnified homogeneous sedimentation of sample14. (**c**). Sample17 showing larger quantity of sediment and Al backing (**d**) Magnified homogeneous sedimentation of sample 17.

The stacked-sample activation technique was used for cross-section measurements of proton-induced reactions on enriched germanium (^72^Ge = 96.42%). Thin metal samples of enriched germanium were prepared at the Forschungszentrum Jülich (FZJ) by the sedimentation technique^23^. The powdered enriched germanium (supplied by Chemotrade GmbH, Germany) was used to develop thin layers on Al foils of 50 μm thickness and 13 mm diameter (supplied by Goodfellow; chemical purity: 99.0%) as backing material of the sediments. About 40 mg enriched germanium metal powder was suspended in 20 mL of toluene containing a very small amount of Levaprene‐450 (obtained from Arlanxeo, Dormagen, Germany) which possesses high adhesive properties, thereby vastly increasing the stability of the sediment and the attachment to the supportive backing. The supportive backing, a 50 μm aluminum foil, was weighed and fitted between two 10 mm thick Teflon discs screwed together. One of the discs contained a hole of 10 mm diameter forming a 0.785 mL cavity on top of the aluminum backing. The suspension containing the enriched germanium metal was mixed thoroughly, before 0.75 mL of this suspension was transferred into the sedimentation cell, as homogenous and as rapid as possible. Thereafter, the sedimentation cell was carefully placed in a drying oven and the toluene was allowed to evaporate at room temperature for 2 days. After confirming complete dryness (no dark stains left in the sediment), the sedimentation cell was carefully unscrewed and the aluminum backing together with the sediment was removed. The sedimented layers were examined under a microscope and only homogeneous and mechanically stable samples were selected for irradiation. Figure 5 shows the pictures of the surface of two typical samples used in this study as the targets. Passing the quality control, an aluminum cover of 15 mm diameter and 10 μm thickness of known weight was placed on top of each sediment and welted around the aluminum backing. Afterwards, the whole package was weighed, and the exact mass of the sediment was calculated. The exclusive weights of the sediments were found in the range of 2–7 mg. Therefrom the area density was calculated for each sample. The thus prepared enriched germanium sediment, sandwiched between two Al foils, served as the target sample.

**Figure 6:**
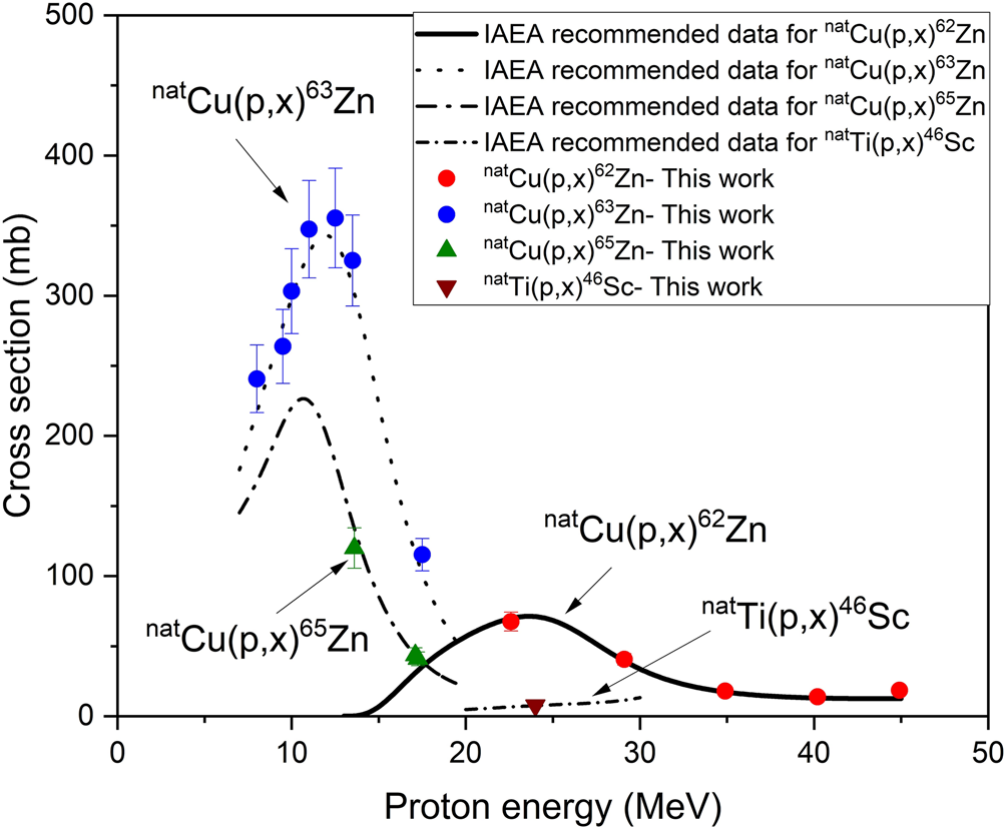
A comparison of the monitor reaction cross sections derived over the investigated energy regions in this work and the IAEA recommended data.

Seven samples were stacked together with four thin circular foils (diameter 13 mm) of Cu and one foil of Ti (supplied by Goodfellow; purity: Cu (99.9%); Ti (> 99.6%) and were irradiated at C30-XP. Similarly, thirteen enriched samples together with seven Cu monitor foils were used for irradiation at JULIC and ten samples together with seven Cu monitor foils were irradiated at BC 1710. The flux obtained from each monitor foil was used for the cross section calculations at the respective energy. The energy on each target foil is listed in Table 5. The thicknesses of Cu foils for the three experiments were chosen according to the energy provided by each cyclotron. These Cu and Ti foils served as beam monitors to determine the proton flux and energy along the whole stack. To decrease the energy of protons on each target, additional copper degraders were used (supplied by Goodfellow; purity: 99.9%; thickness: 250 μm and 110 μm). In total three stacks, each with different number of samples, for three different cyclotrons together with several monitor foils, were irradiated. The first stack was irradiated at Jülich Isochronous Cyclotron (JULIC) up to 45 MeV: the second at the BC 1710 cyclotron, and the third at C30-XP. The stacks irradiated at the BC 1710 were mounted in the screw-capped dummy target holder of the beam-line extension constructed a few years ago^33^. The JULIC is an old established machine at FZJ. It has been in use for many years as the injector of the high-energy cooler synchrotron (COSY). Recently an external target station was constructed at JULIC with an adapter where several target holders can be fitted. In the present measurements, the standard screw-capped dummy target holder, similar to the one at BC 1710^34^, was used to mount the samples and foils in a stacked-form. The beam characterization and beam flux monitoring for the experiments at BC 1710 and JULIC, with protons of primary energies of 16.7 MeV and 45.0 MeV have already been reported. The irradiation at the C30-XP was carried out on a new target system. This is a new machine and is being tuned both for cross section measurements and clinical scale radionuclide production. Some details on beam characterization and flux determination during the present experiment are given here. The extracted beam is of high precision with respect to energy and profile definition. Nevertheless, we checked the effective energy of the proton beam in a foil by the activity ratio method^33,34^. The ^63^Zn/^62^Zn activities formed in Cu monitor foils, mounted in the stack, were considered for determining the accuracy of energy. The activities of these radionuclides were determined non-destructively by γ-ray spectrometry, converted to decay rates with necessary corrections, and finally extrapolated to the end of bombardment (EOB). The mean energy of the proton beam in the front Cu foil as well as in the subsequent foils was determined by comparing the experimentally obtained ratios with the values calculated theoretically from the IAEA recommended excitation functions of the reactions ^nat^Cu(p,x)^63^Zn (energy range 7–20 MeV), ^nat^Cu(p,x)^62^Zn (energy range 20–45 MeV)^35^. The deduced energy agreed well with the value derived from the accelerator parameters. Keeping the uncertainties of the cross sections in mind we fixed the primary energy of the extracted proton beam of the cyclotron as 24.2 ± 0.3 MeV. The effective proton flux was determined by using the reactions ^nat^Cu(p,x)^62^Zn, ^nat^Cu(p,x)^65^Zn (energy range 7–20 MeV) and ^nat^Ti(p,x)^46^Sc (energy range 20–30 MeV), as monitor reactions. A comparison of the monitor reaction cross sections over the investigated respective energy regions derived in this work and the recommended data of the IAEA is given in Fig. 6. The data fits very well within the limits of uncertainties. Those monitor reactions were chosen owing to their nearly stable excitation function and better recommended precision over the proton energy range of this work. The proton flux was determined using the measured decay rates of ^62^Zn, ^65^Zn and ^46^Sc at EOB and the recommended cross-section values of the respective monitor reactions^35^. The proton flux values from the above three monitors agreed with the average value within 2–6%. The energy degradation was calculated by the computer program, STACK, written at FZJ and based on the energy-range relation^36^.

**Table 5:** Measured cross sections for the formation of ^71^As and ^72^As in proton irradiation of enriched ^72^Ge at three cyclotrons at FZJ.

Proton energy (MeV)	Cross section (mb) of ^72^As	Cross section (mb) of ^71^As
Cyclotron JULIC		
44.6 ± 0.3	42.4 ± 5.5	47.2 ± 6.1
42.5 ± 0.3	48.1 ± 6.2	27.5 ± 3.6
40.0 ± 0.3	44.2 ± 5.3	47.3 ± 6.1
37.7 ± 0.4	22.7 ± 2.7	50.2 ± 6.5
34.9 ± 0.4	28.6 ± 3.4	73.8 ± 9.6
32.3 ± 0.4	29.3 ± 3.5	97.8 ± 12.7
29.2 ± 0.4	33.2 ± 4.0	138.8 ± 18.0
26.2 ± 0.4	44.5 ± 5.3	222.0 ± 28.8
23.1 ± 0.4	60.6 ± 7.3	323.4 ± 42.0
20.1 ± 0.4	113.4 ± 13.6	363.3 ± 47.2
16.0 ± 0.5	350.1 ± 42.0	200.4 ± 26.0
13.7 ± 0.5	649.7 ± 77.9	34.5 ± 4.5
11.1 ± 0.5	713.0 ± 85.5	
Cyclotron BC-1710		
16.4 ± 0.2	337.8 ± 37.2	283.5 ± 34.0
16.0 ± 0.2	419.3 ± 46.1	263.8 ± 31.6
13.7 ± 0.2	694.5 ± 76.4	6.6 ± 0.8
12.5 ± 0.2	669.5 ± 73.6	
11.7 ± 0.3	712.2 ± 78.3	
10.8 ± 0.3	751.4 ± 82.6	
10.2 ± 0.3	615.3 ± 67.7	
9.3 ± 0.3	546.4 ± 60.1	
8.3 ± 0.4	502.9 ± 55.3	
7.3 ± 0.4	409.7 ± 45.1	
Cyclotron C30-XP		
24.2 ± 0.3	52.8 ± 6.3	317.6 ± 41.3
20.3 ± 0.3	108.1 ± 12.9	399.1 ± 51.9
16.4 ± 0.4	347.3 ± 41.7	196.5 ± 25.5
16.0 ± 0.4	418.0 ± 50.2	167.8 ± 21.8
14.6 ± 0.4	631.8 ± 75.8	39.8 ± 5.2
13.2 ± 0.5	654.9 ± 78.6	
12.1 ± 0.5	706.6 ± 84.8

### Radioactivity measurement

**Figure 7:**
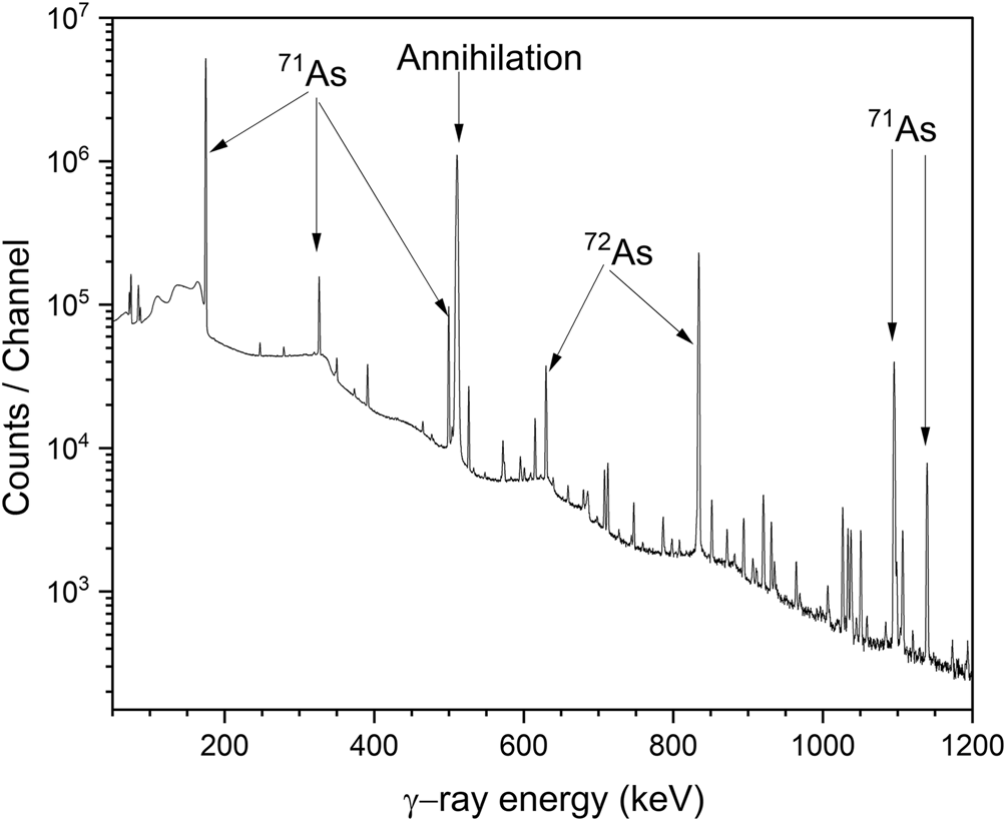
Typical γ-ray spectrum of an enriched ^72^Ge sample irradiated with 24 MeV protons. The most important peaks originating from ^72^As and ^71^As (given in Table 2) are marked in the diagram.

The radioactivity of a relevant radionuclide formed in irradiated enriched germanium-sample or in monitor foil was measured nondestructively using several high-purity germanium (HPGe) gamma-ray detectors, supplied by ORTEC, coupled with the necessary electronics and Maestro data acquisition software. The energy resolutions (FWHM) of the detectors used at 1332.5 keV of ^60^Co were 1.9–2.5 keV. The standard point sources ^22^Na, ^54^Mn, ^57^Co, ^60^Co, ^137^Cs, ^152^Eu, and ^241^Am, supplied by Eckert and Ziegler, Berlin, were used for efficiency calibration of the γ-ray detectors. The uncertainty in the activity of each source was specified as 3%. The γ -ray spectra measured were analyzed by the GammaVision^37^ software. A typical gamma ray spectrum is shown in Fig. 7. The major γ-rays from ^72^As and ^71^As are labeled. The background was subtracted to get the net area for each γ -ray. Samples were counted at various distances, viz. 10, 20, 30 and 50 cm from the surface of the detector, depending on the half-life and activity of the irradiated sample. The dead time of the system was kept below 5%. For all the above counting distances, the effect of the sample size on the efficiency and also the random coincidence loss became almost negligible. Measurements were carried out in several parallel steps, depending on the half-life of the product. The activity of short-lived radionuclides was measured within 2–3 h after EOB. Special attention was paid to attenuation of γ -ray as well as to detector efficiency for that gamma line. During the counting the Al-cover (10 μm thick) side of the germanium-sediment was always kept downward, i.e., facing the detector to minimize the absorption in Al. The further absorption of the low energy gamma lines in both the sample and the Al-cover was estimated; it amounted to about 0.05% and 0.03%, respectively. Parallel to this measurement, the monitor foils were also measured on the other detectors whose efficiencies were known for the given distances. In the second step of counting, the radioactivity of each of the two radionuclides ^72^As (T_1/2_ = 26.0 h) and ^71^As (T_1/2_ = 65.30 h) was determined.

### Experimental cross sections

The experimental nuclear reaction cross sections were measured using thin samples of ^72^Ge metal in the stacked-sample activation arrangement covering the energy range from 7.3 to 45 MeV. The beam profile and flux measurements were carried out by using Cu and Ti monitor foils. The production cross sections for the monitor reactions are well-established as described above. A photograph of two typical thin samples of enriched ^72^Ge (Sample 14, Sample 17), prepared in our laboratory, is shown in Fig. 5. Irradiations were done at three different cyclotrons at FZJ. The radioactivity of each product was determined via high-resolution γ-ray spectrometry. A typical γ-ray spectrum of an enriched Ge sample irradiated with 24 MeV protons is shown in Fig. 7. Further details of the measurements are given above in the section Methods and Analytical Procedures. The measured cross sections and the associated uncertainties are summarized in Table 5. In a few samples irradiated at JULIC, at proton energies above 35 MeV a very weak activity of ^70^As (T_1/2_ = 52.6 min) formed via the ^72^Ge(p,3n)^70^As reaction was also detected. However, due to rather poor counting statistics we did not analyse its production cross section.

### Nuclear reaction cross section calculations

Nuclear reaction cross sections were calculated by using the three models, namely TALYS, EMPIRE and ALICE-IPPE. The nuclear model code TALYS^26^ version 1.96 was used, adopting an equidistant excitation energy grid. TALYS 1.96 consists of several nuclear models to analyze all the possible nuclear reaction mechanisms over the energy range of 1 keV to 200 MeV. In the calculations, considerable parametrisation was done in an attempt to obtain good agreement with the experimental data. The particle transmission coefficients were generated via the spherical optical model by using the ECIS-06 code^38^ with global parameters: for neutrons and protons from Koning and Delaroche^39^; for the optical model parameters (OMP) of complex particles (d, t, ^3^He) the code made use of a folding approach, building up the OMPs from the neutron and proton potential. The proton optical model was adjusted by using the recommended range of the parameter (*rvadjust* = 0.9). For OMP of alpha particles the TALYS default reference parameter set was adopted. Width fluctuation calculations were carried out for the compound nuclear part of the excitation function. The gamma-ray transmission coefficients were calculated through the energy-dependent gamma-ray strength function according to Kopecky and Uhl^40^ for E1 radiation, and according to Brink^41^ and Axel^42^ for all the other transition types. For the pre-equilibrium contribution in the nuclear reactions, a two-component exciton model of the TALYS code was used. The collective contributions from giant resonances and surface effects in exciton model were also calculated. The onset energy for multiple pre-equilibrium processes was set to 20 MeV. The energies, spins, parities, and branching ratios of the discrete levels were based on the RIPL-3 database^43^. The spin cutoff factor for the ground state was set equal to 1. The vibrational enhancements and shell correction energies were taken into account for calculations. In the continuum region, the level density was calculated by the geometry dependent superfluid model (GDSFM)^44^, using its slightly modified new version in TALYS 1.96. The basic physical and numerical parameters and the reaction mechanisms are given in the manual of TALYS 1.96. The calculated results from the global library of TALYS, i.e. TENDL-2021^27^ were also compared. The detailed description of adopted parameters and all analytical nuclear model calculations are given in the manual of TALYS 1.96 that is available at the official website of the International Atomic Energy Agency (IAEA). (iaea.org)^45^

For comparison, another nuclear model code EMPIRE 3.2 was also employed. The code was developed by Herman et al.^25^ under international cooperation. The calculations were done by using the discrete levels taken from the RIPL-3 level file, based on the 2007 version of ENSDF. EMPIRE specific level densities were used and exciton model calculations were done with PCROSS. The cluster emissions given by Iwamoto-Harada model were adopted. Monte Carlo preequilibrium model (HMS)^44^ was used by assuming the isotropic angular distribution of the compound nucleus.

For purely precompound model calculations ALICE-IPPE was used that contains the generalized super fluid model for level densities.

The cross section calculations for the production of ^72^As and ^71^As by using the above mentioned three nuclear model codes as well as TENDL-2021 data are compared and discussed above with the experimental data.

#### Uncertainties

The total uncertainty in the cross sections measured in the present work was estimated by the sum of both statistical and systematic errors^46^. The statistical errors included in each cross-section value contain the individual uncertainties in: counting statistics (1–5%), true coincidence correction (< 2%), decay data, especially γ-ray intensities (1.9–8.4%) and sample homogeneity (up to 5%). The uncertainty of the net peak area was (1.5–5%), obtained from the analysis of the weak and unsmooth peaks, from where, the uncertainty associated with the peak area was calculated manually. The uncertainty in the measurement of the beam flux varied for the three cyclotrons (3–6%). The systematic errors include the detector efficiency (2.5–5.5%), nuclear data (5–6%), and reference cross sections (4–5%). The overall total uncertainty in the cross sections for ^72^As and ^71^As is estimated as (11–13%). The energy uncertainty of the proton beam also varied (2–5%) for the three cyclotrons that became gradually higher as the beam got degraded in each foil.

## Data Availability

The datasets used and/or analysed during the current study are available from the corresponding author on reasonable request.
